# Improving Web-Based Treatment Intake for Multiple Mental and Substance Use Disorders by Text Mining and Machine Learning: Algorithm Development and Validation

**DOI:** 10.2196/21111

**Published:** 2022-04-11

**Authors:** Sytske Wiegersma, Maurice Hidajat, Bart Schrieken, Bernard Veldkamp, Miranda Olff

**Affiliations:** 1 Department of Research Methodology, Measurement and Data Analysis University of Twente Enschede Netherlands; 2 Interapy PLC Amsterdam Netherlands; 3 Department of Psychiatry Amsterdam University Medical Centres, location Academic Medical Centre Amsterdam Netherlands; 4 ARQ National Psychotrauma Centre Diemen Netherlands

**Keywords:** supervised text classification, multi-class classification, screening, mental health disorders, computerized CBT, automated intake and referral

## Abstract

**Background:**

Text mining and machine learning are increasingly used in mental health care practice and research, potentially saving time and effort in the diagnosis and monitoring of patients. Previous studies showed that mental disorders can be detected based on text, but they focused on screening for a single predefined disorder instead of multiple disorders simultaneously.

**Objective:**

The aim of this study is to develop a Dutch multi-class text-classification model to screen for a range of mental disorders to refer new patients to the most suitable treatment.

**Methods:**

On the basis of textual responses of patients (N=5863) to a questionnaire currently used for intake and referral, a 7-class classifier was developed to distinguish among anxiety, panic, posttraumatic stress, mood, eating, substance use, and somatic symptom disorders. A linear support vector machine was fitted using nested cross-validation grid search.

**Results:**

The highest classification rate was found for eating disorders (82%). The scores for panic (55%), posttraumatic stress (52%), mood (50%), somatic symptom (50%), anxiety (35%), and substance use disorders (33%) were lower, likely because of overlapping symptoms. The overall classification accuracy (49%) was reasonable for a 7-class classifier.

**Conclusions:**

A classification model was developed that could screen text for multiple mental health disorders. The screener resulted in an additional outcome score that may serve as input for a formal diagnostic interview and referral. This may lead to a more efficient and standardized intake process.

## Introduction

### Background

Mental and substance use disorders such as anxiety, mood, alcohol and drug use, eating, and depressive disorders have been listed among the leading causes of global disability over the past years [1]. Annual studies show that between 2010 and 2016, these disorders accounted for approximately 18%-19% of the global burden of disease, measured in years lived with disability [2]. The proportion of people living with a mental disorder has remained practically unchanged in recent years (approximately 15.6%, 17.6%, and 19% for the global, European, and Dutch populations, respectively). However, because of population growth, absolute numbers of people diagnosed with a mental disorder have increased by 72 million globally and by 2 million in Europe between 2010 and 2016. For the Netherlands, despite an initial decrease in numbers by 15,000 from 2010 to 2014, there was an increase by 4000 between 2014 and 2016.

This growing number of people requiring mental health care each year makes preventing and detecting mental disorders, implementing early interventions, and improving treatments and mental health care access to public health and research priorities [3,4]. Mental health disorders are usually treated through medication or psychotherapy such as cognitive behavioral therapy (CBT), of which psychotherapy is generally seen as the first-line treatment [5]. However, mental health treatments are often underused [6] or delayed for many years [7]. Especially in low- and middle-income countries, there is a huge treatment gap in mental health care; 75% of the people experiencing anxiety, mood, impulse control, or substance use disorders remain untreated [8]. The reasons for this could be individual patient factors (eg, embarrassment, lack of time, and geographic influences); provider factors (eg, underdetection and lack of skill in treating mental health problems); or systemic factors such as limited access to, or limited availability of, mental health providers, resulting in waiting lists [6].

This calls for more efficient, accurate, and accessible screening and treatment methods [9,10]. Modern technologies are increasingly recognized as a means of improving the accessibility of care and advancing the assessment, treatment, and prevention of mental health disorders. Creative, low-cost approaches should be used to increase access to (trauma-focused) CBT and other treatments [11]. An example of such an approach is web-based self-help, which is an increasingly available alternative for a range of disorders. Web-based self-help can be therapist-guided or not, and although some studies reported equal effects for guided and unguided web-based treatment (eg, for social anxiety disorders [12] and depression [13]), most research endorses the importance of at least minimal, regular therapist guidance in psychological interventions [14,15]. Web-based therapist-guided treatment such as computerized CBT is found to be approximately as effective as face-to-face treatment for several mental health disorders (eg, depression, anxiety, and burnout) [16-18].

In the Netherlands, 1 party offering web-based, therapist-assisted CBT is Interapy, a web-based mental health clinic approved by the Dutch health regulatory body. Interapy conducts screening, treatment, and outcome measurement on the web. Patient intake and diagnosis is performed using validated self-report instruments, followed by a diagnostic interview by telephone, after which patients are referred to a protocolled disorder-specific treatment. The treatment consists of a fixed set of evidence-based homework assignments provided through the Interapy platform and uses standardized instructions that are tailored to the patient by a therapist. After submitting the homework assignments, the patient receives asynchronous personal feedback and new instructions [14].

This form of web-based therapy generates large quantities of digital text data to be processed manually by the treating therapist. Textual data contain a lot of information that could be used more efficiently in the screening and treatment process through the application of text mining techniques. Text mining is generally used to automatically explore patterns and extract information from unstructured text data [19]. There is a large body of literature on text mining applications in the field of psychiatry and mental health; 2 recent systematic literature reviews provide a useful overview of the scope and limits, general analytic approaches, and performance of text mining in this context [20,21]. Abbe et al [20] concluded that text mining should be seen as a key methodological tool in psychiatric research and practice because of its ability to deal with the ever-growing amount of (textual) mental health data derived from, for example, medical files, web-based communities, and social media pages. However, despite the amount of data that are generated, assembling large, high-quality mental health text data sets has been found to be difficult [21]. With regard to the analytic approach, in most studies, predictive models are developed using supervised learning algorithms such as support vector machines (SVMs) and verified using *k*-fold cross-validation [21].

A way in which text mining can be put to use in mental health care practice concerns the detection of mental disorders. Previous studies showed that text mining can be used successfully in screening for posttraumatic stress disorder (PTSD) and depression [22,23]. He et al [22] developed an automatic screening model for PTSD using textual features from self-narratives posted on a forum for trauma survivors. On the basis of a set of highly discriminative keywords and word combinations extracted from the narratives using text mining techniques, they developed a text classifier that could accurately distinguish between trauma survivors with and those without PTSD. They concluded that automatic classification based on textual features is a promising addition to the current screening and diagnostic process for PTSD that can be easily implemented in web-based diagnosis and treatment platforms for PTSD and other psychiatric disorders. Neuman et al [23] developed an automatic screening system for depression using a *depression lexicon* based on metaphorical relations and relevant conceptual domains related to depression harvested from the internet. This lexicon was used to screen texts from open questions on a mental health website and a set of general blog texts for signs of depression and was found to classify texts that included signs of depression very accurately.

Although both studies showed the technical potential of automatic text classification in screening for mental disorders, they applied a proxy or a self-reported diagnosis instead of a direct, formal diagnosis by a psychiatrist as the classification criterion. In addition, both studies developed a binary classifier that focused on recognizing only a single specific disorder (PTSD or depression) at a time, which is the case in most studies that apply text mining to detect mental disorders [20,21]. However, in practice, for many patients who register with mental health complaints or sign up for web-based treatment, it is not clear beforehand which disorder they should be screened for. In this case, a multi-class classifier, screening for multiple different mental disorders at once, would be more useful than a binary classifier screening for only a single prespecified disorder. Finally, it is pointed out that most natural language processing tools are currently designed for exploring English texts [20]. Although, indeed, text mining and language processing tools are mainly developed for the English language, the methods and techniques underlying the text analysis process are not necessarily language dependent. The development of models for different languages depends mainly on the availability of training and testing corpora and not so much on the methods and techniques used, as will be demonstrated in this study.

### Objectives

This study investigates if and to what extent automatic text classification can improve the current web-based intake procedure of a Dutch web-based mental health clinic. The current intake questionnaire (see *Methods* section) consists of open and multiple-choice questions. The multiple-choice answers are converted to scores on four scales (somatization, depression, distress, and anxiety) as well as estimates of symptom severity, required level of care, suicide and psychosis risk, and drug dependence. These scores lead to an automatically generated indicative referral advice. This advice and the answers to the open questions are used by the therapist as input for the subsequent diagnostic telephone interview to arrive at a formal diagnosis and referral advice. However, the current questionnaire does not cover all disorders for which treatment is offered by Interapy, and the textual answers to the open questions remain to be processed and interpreted by the therapist. An automatic text screener may provide the therapist with more specific additional information, making the intake process more efficient and standardized.

Therefore, a multi-class text-classification model has been developed to screen for a range of different mental disorders with the aim of referring newly registered patients to the most fitting treatment. The focus is on a selection of treatments currently offered by Interapy for anxiety and panic disorders, PTSD, mood disorder (including depressive disorders), eating disorder, substance use disorders, and somatic symptom disorders. These will be referred to, respectively, as *Anxiety*, *Panic*, *PTSD*, *Mood*, *Eating*, *Addiction*, and *Somatic* throughout the rest of this paper. The treatment choice was made based on the amount of text data that was readily available from the Interapy database at the time of this research. This study adds to existing research in that (1) the patients in our sample have an official clinical diagnosis made by a therapist; (2) our data set consists of patients with a variety of mental health disorders, enabling us to develop a multi-class text classifier; and (3) the derived texts and the resulting classifier are in Dutch and as such provide an example of non-English text mining efforts applied in mental health care research and practice.

## Methods

### Methods Overview

The multi-disorder screening model was developed based on text and questionnaire data collected through the web-based intake environment of Interapy. This section describes the methods and techniques used to develop the supervised text-classification model and evaluate its performance.

### Data Set

We used pretreatment scores on a self-reported questionnaire and text data derived from 3 open questions collected within the web-based intake environment. The patients are Dutch adults and adolescents who were referred to one of Interapy’s web-based treatments by their general practitioner and diagnosed by a therapist. All participants have given permission for their treatment data to be used for anonymized research by Interapy to improve and evaluate their treatments through informed consent. The electronic patient database was queried in July 2017. For each treatment, all available data were retrieved, excluding incomplete or double entries. For treatments for which large quantities of data were available, a random sample of 1100 patients was drawn to distribute the available data across the classes more evenly.

### Web-Based Questionnaire

After signing up, new patients were asked to fill in the Digitale Indicatiehulp Psychische Problemen (DIPP; Digital Indication Aid for Mental Health Problems) questionnaire, an approved and validated decision support tool developed by Interapy and the HSK group, a national organization for psychological care in the Netherlands [24,25]. The DIPP questionnaire consists of the Dutch version [26] of the Four-Dimensional Symptom Questionnaire [27,28], complemented with several multiple-choice and open questions. The 4D Symptom Questionnaire contains 50 multiple-choice questions measuring distress, depression, anxiety, and somatization, which are dimensions of common psychopathology [27]. The complementary questions relate to current symptoms, treatment goals, anamnesis, psychosis risk, substance use, and medication. The DIPP questionnaire was originally developed, validated, and published in Dutch. A translated version of the questionnaire is provided in Multimedia Appendix 1. The answers to the following three open questions were used to develop the text-classification model:

1. Can you briefly describe your main symptom or symptoms?
2. What would you like to achieve with a treatment?
3. Have there been any events (such as a divorce, loss of job, or accident) that, in your opinion, affect your current symptoms, and if so, what are they?

The information collected through the DIPP questionnaire results in scores on four scales: somatization, depression, distress, and anxiety. Each patient is then assigned a weight to indicate symptom severity and level of care (no care, general practice mental health care, basic mental health care: short, basic mental health care: moderate, basic mental health care: intensive, and specialist mental health care). The outcome is verified by a semistructured diagnostic interview over the telephone, which results in a formal referral advice and diagnosis. Intake, diagnosis, referral, and treatment are all conducted by a CBT-certified health psychologist.

### Automated Text-Screening Model

#### Supervised Classification

**Figure 1:**
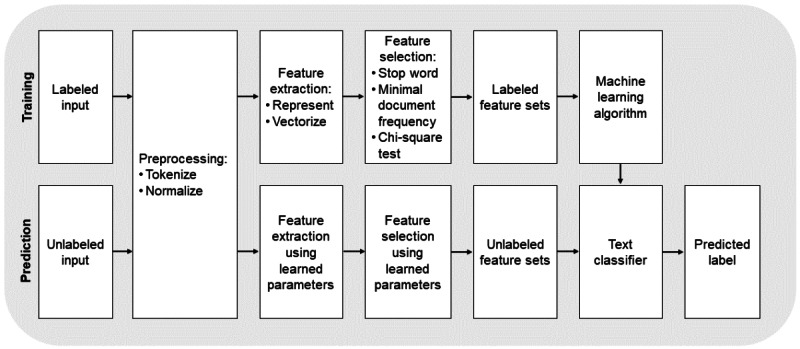
Supervised text classification model procedure. In the training phase, the model is trained on labeled feature sets extracted from the input texts. In the prediction phase, the trained model is used to predict labels for new, unlabeled feature sets extracted from the input texts.

To screen future textual answers on the 3 open questions of the DIPP questionnaire for the presence of anxiety and panic disorders, PTSD, mood disorders, eating disorders, substance addiction, or somatic symptom disorders, a supervised multi-class text classifier was developed. It is called a supervised classifier because it was developed based on an existing set of text fragments provided with the correct diagnostic labels. The answers to all 3 questions were combined into 1 text document per patient. The formal referral advice based on the DIPP questionnaire scores and the diagnostic interview was used as the diagnostic label to be predicted by the model. The classifier is multi-class because the model refers each input text to 1 of multiple classes: the 7 disorders present in the input corpus. The development of a supervised classification model follows a 2-phase strategy: a model-training phase and a label-prediction phase. This section explains the steps taken in each phase. The complete classification procedure is shown graphically in Figure 1.

#### Training

During training, text features (words or word combinations) are extracted from each input text, converting the texts to labeled feature sets. These labeled feature sets are used as input for the machine learning algorithm, which generates a multi-class model by selecting the most informative features for each class.

#### Preprocessing

Standard preprocessing steps such as tokenization (splitting texts into separate tokens such as words, numerical expressions, and punctuation) and normalization (removing punctuation, converting capital letters to lowercase letters, and stripping off accents) were applied to process all texts at the word level [29]. All words were brought back to their core, meaning-baring stem using the Snowball Stemmer, a standard stemming algorithm available for many languages, including Dutch [30]. The resulting set of words for each input text is termed the vocabulary and consists of tokens, all used words or word combinations, and types, all unique words or word combinations used [31].

#### Feature Extraction

To convert the resulting vocabularies to feature sets suitable as input for the machine learning algorithm, the dimensionality of the feature space was reduced by feature extraction and feature selection techniques. For feature extraction, different document representation and vectorization schemes were compared. The document representations considered were unigrams, *N*-grams, and *N*-multigrams, which are single words, sequences of *N* words, and variable-length sequences of maximum *N* words, respectively [32]. The vectorization schemes refer to the specified term weights, for which we used normalized term frequency [33] or term frequency–inverse document frequency [34].

#### Feature Selection

Stop word removal, minimal document frequency, and the Pearson chi-square test were used to select the most informative features. Stop word removal was considered because stop words are generally not expected to contribute to the meaning of the text [29], although other studies contradict this [35]. In addition, words that only occur sparsely throughout the complete corpus (document frequency) may also be removed [36]. The most informative features (features with the highest chi-square values) are found by ranking features based on their Pearson chi-square value, a common and highly efficient method that measures the independence among corpora by comparing the observed and expected feature occurrences in each class [33]. The optimal number of features to select is determined by an exhaustive parameter grid search, which will be further explained in the section *Analytical Strategy*.

#### Machine Learning Algorithm

The selected features and their corresponding labels from the training set form the labeled feature sets that were used as input for the machine learning algorithm. The SVM [37] was used because this is a high-performing and robust classification algorithm that deals well with high-dimensional data such as text [36]. As SVMs were originally intended for binary classification tasks, multi-class (*K*-class) classification tasks were split into *K* binary classification tasks following the one-against-all (O-a-A, also known as one-versus-rest) or the one-against-one (also known as one-versus-one) decomposition strategy.

The one-against-one strategy, which compares each pair of classes separately [38,39], is generally considered a better approach when dealing with class imbalance, as was present in our data set. However, this strategy requires substantially more computational resources because many pairwise SVMs need to be trained. We therefore applied the widely used O-a-A strategy, which compares each single class with the remaining classes [38,39]. This strategy is the most commonly used, thanks to its computational efficiency and interpretability. To compensate for the class imbalance, a class-weighting scheme was used where classes were weighted to be inversely proportional to the class frequencies in the complete data set (as proposed by King and Zeng [40]). This puts more emphasis on the information extracted from the smaller classes and prevents the highly present classes from overshadowing the classification model.

The SVM with O-a-A strategy was implemented in the linear support vector classifier within the LIBLINEAR library developed by Fan et al [41]. Finally, 2 hyperparameters could be optimized for the SVM model: the kernel parameter *γ* [42], which controls model flexibility [43], and the regularization parameter *C*, which controls training and testing error [42]. We used a linear kernel as is common in text classification [36] and optimized the regularization parameter in the grid search (see *Analytical Strategy*).

#### Prediction

During prediction, text features of new, unlabeled input texts were extracted and converted to feature sets following the same strategy used during training. Following the O-a-A approach, we fitted 7 SVMs, 1 for each disorder, alternately comparing 1 of the 7 classes (the positive class) to the remaining 6 (together forming the negative class). As described by James et al [44], this results in 7 separate binary classification models, each with their own parameters *β*_0k_*,β*_1k_*,...,β_pk_*, with *k* denoting the *k^th^* class and *p* the number of learned parameters. Each new, unlabeled input text *x* was provided with the class label for which the confidence score *β*_0k_+*β*_1k_*x*_1_+*β*_2k_*x*_2_+···+*β_pk_x_p_* was the largest. This showed that there was a high level of confidence that the input text belonged to this class and not to one of the other 6 classes.

#### Confusion Matrix

The performance of the classifier was measured by comparing the predicted labels with the known labels for each class using a confusion matrix. A confusion matrix displays the instances in the predicted classes per column and the true classes per row, directly visualizing the number of correctly labeled documents on the diagonal and the errors (mislabeled documents) in the surrounding cells [31]. Table 1 shows the confusion matrix for a 7-class classifier with classes *A-G*.

The number of true positives for class *A* (*TP_A_*) were the number of times a document was labeled with *A* and the true label was indeed *A*. The false positives for class *A* (*FP_A_*) were the instances that were incorrectly labeled by the classifier as *A*, whereas the true label was not *A*. This was calculated for class *A* by using the following formula:

*E_B,A_*+*E_C,A_*+*E_D,A_*+*E_E,A_*+*E_F,A_*+*E_G,A_*

The false negatives for class *A* (*FN_A_*) were the instances with true label *A* for which the classifier predicted a different label. This was calculated for class *A* by using the following formula:

*E_A,B_*+*E_A,C_*+*E_A,D_*+*E_A,E_*+*E_A,F_*+*E_A,G_*

**Table 1:** Confusion matrix for the 7-class classifier: comparison of true and predicted class labels for classesA-G.

True label	Predicted label						
	Class_A_	Class_B_	Class_C_	Class_D_	Class_E_	Class_F_	Class_G_
Class_A_	*TP*_A_^a,b^	E_A,B_	E_A,C_	E_A,D_	E_A,E_	E_A,F_	E_A,G_
Class_B_	E_B,A_	*TP*_B_^b^	E_B,C_	E_B,D_	E_B,E_	E_B,F_	E_B,G_
Class_C_	E_C,A_	E_C,B_	*TP*_C_^b^	E_C,D_	E_C,E_	E_C,F_	E_C,G_
Class_D_	E_D,A_	E_D,B_	E_D,C_	*TP*_D_^b^	E_D,E_	E_D,F_	E_D,G_
Class_E_	E_E,A_	E_E,B_	E_E,C_	E_E,D_	*TP*_E_^b^	E_E,F_	E_E,G_
Class_F_	E_F,A_	E_F,B_	E_F,C_	E_F,D_	E_F,E_	*TP*_F_^b^	E_F,G_
Class_G_	E_G,A_	E_G,B_	E_G,C_	E_G,D_	E_G,E_	E_G,F_	*TP*_G_^b^

^a^TP: true positive.

^b^The values on the diagonal (in italics) show the correctly predicted class labels. The off-diagonal values show the prediction errors.

#### Performance Metrics

The correct predictions (TPs and TNs) and errors (FPs and FNs) were then used to calculate performance metrics for each class. Bird et al [31] define several metrics, the simplest of which is accuracy, a measure for the proportion of correctly labeled input texts in the test set. The recall, also called sensitivity or TP rate, indicates how many of the text documents with a true (known) positive label were identified as such by the classifier and is calculated for each class by using the following formula: TP*/*(TP+FN). The precision (also known as positive predictive value) is calculated for each class by using the formula TP*/*(TP+FP) and concerns the proportion of positively predicted text documents where the true (known) label was indeed positive. The harmonic mean of the precision and recall, 2 × (*Precision*×*Recall*)/(*Precision*+*Recall*), is the *F*_1_ score. The overall performance scores for the classifier were calculated by averaging the performance scores of all classes (ie, all 7 binary SVMs that were fitted following the O-a-A approach). We used weighted macroaveraged scores because this accounts for class imbalance; as this method gives equal weight to each class, it prevents the most occurring classes from dominating the model [45].

### Analytical Strategy

**Figure 2:**
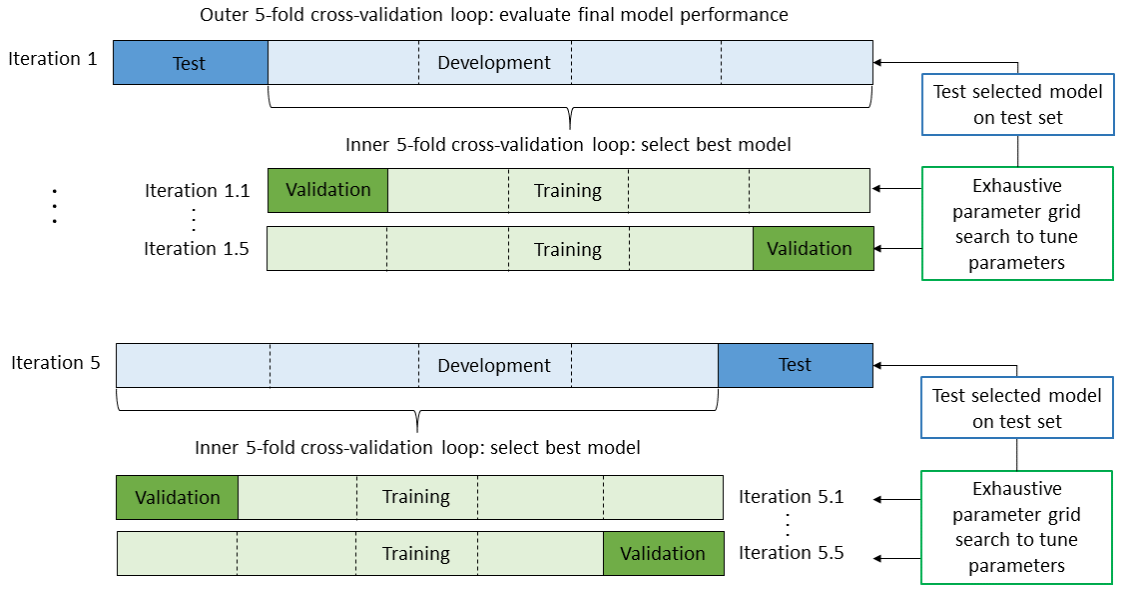
Nested 5-fold cross-validation scheme. The validation strategy consists of an inner and an outer 5-fold cross-validation loop. In the inner loop an exhaustive parameter grid search is conducted using data from the development set to select the best combination of parameter settings. The selected model is then tested on the held-out test set from the outer loop to evaluate final model performance. Both loops are being iterated 5 times, alternately using each fold as test set (outer loop) or validation set (inner loop) once.

To prevent model evaluation bias, different subsets of the data were used to train, validate, and test the model. A nested *k*-fold cross-validation strategy was adopted, using a 5-fold cross-validated grid search in the inner loop for model selection and 5-fold cross-validation in the outer loop for model evaluation (see Figure 2 for a schematic representation). To make sure all classes were represented in each fold in approximately the same proportions as in the complete data set, stratified sampling [46] was used in both cross-validation loops.

For the outer loop, the data set was first split into 5 folds, alternately defining 4 folds as the development set for model selection and setting aside 1 fold as a test set for assessing final model performance and generalization. To optimize the different model parameters, an exhaustive parameter grid search was conducted in the inner loop. In this grid search, all possible combinations of parameter values were fitted on the data set in search of the combination resulting in the highest performance score. The following model parameters and parameter values were compared:

- Choice of representation scheme: unigrams, bigrams, trigrams, or 3-multigrams
- Term weights: term frequency or term frequency–inverse document frequency
- Stop words: included or excluded
- Minimal document frequency: 1, 2, 3
- Optimal number of features: ranging from 1 to 500, increasing with steps of 20
- Regularization parameter *C*: 1, 2, 3, 10, 100, 1000

The search can be guided by any performance metric. We used the *F*_1_ score because this is the preferred metric when working with imbalanced data sets. The grid search also uses a 5-fold cross-validation approach, splitting the development set into 5 folds, alternately using 4 folds for training and the remaining fold for validation. This is repeated until every fold has been used as the validation set once. The parameter combination that resulted in the highest mean weighted *F*_1_ score over all validation sets was selected as the final model. The generalization performance of the selected model was estimated by again calculating the mean weighted *F*_1_ score, but this time over all test sets from the outer cross-validation loop.

### Text-Classification Tool

The process of model development by means of nested stratified *k*-fold cross-validated grid search is fully automated in a blind text-classification tool developed by the authors. This tool can be used to develop and test a text-classification model on any available text data set without human insight into the data set (hence *blind*). It can be installed and used locally. After installation, no external packages are required; therefore, there is no need to send sensitive information over the internet for external text processing or analysis. An extensive description of the tool, the model development process, and the results on different test data sets will be published in a forthcoming paper by the authors. The tool was applied and described previously in a master’s thesis [47].

### Ethics Approval

This study was approved by the Behavioral, Management, and Social Sciences Ethics Committee of the University of Twente (approval number 220089).

## Results

### Data Set

Table 2 shows the demographic characteristics and DIPP questionnaire results of the patients and the lexical characteristics of their documents for each class. The class labels are *Addiction* (substance use disorders), *Panic* (anxiety disorders with panic attacks), *Anxiety* (anxiety disorders without panic attacks), *PTSD*, *Mood* (mood disorders, including depressive disorders), *Eating* (eating disorders), and *Somatic* (undifferentiated somatoform and other somatic symptom disorders).

**Table 2:** Patient and lexical characteristics (N=5863).

Variable				Addiction (n=197)			Anxiety (n=1100)			Panic (n=1100)			PTSD^a^ (n=1016)		Somatic (n=1100)				Mood (n=1100)			Eating (n=250)				Total (N=5863)		
**Demographic characteristics**																												
	**Gender, n (%)**																											
		Female			18 (9.14)			362 (32.91)			394 (35.82)	498 (49.02)				500 (45.45)				265 (24.09)			180 (72)				2217 (37.81)	
		Male			34 (17.26)			176 (16)			174 (15.82)	119 (11.71)				197 (17.91)				166 (15.09)			8 (3.20)				874 (14.91)	
		Unknown^b^			145 (73.60)			562 (51.09)			532 (48.36)	399 (39.27)				403 (36.64)				669 (60.82)			62 (24.80)				2772 (47.28)	
	Age (years), mean (SD)		37.9 (15.0)			36.5 (14.2)			36.3 (13.8)			36.5 (13.1)					41.2 (11.7)	39.2 (14.4)			30.8 (10.0)			37.7 (13.6)				
**DIPP^c^ questionnaire results: 4DSQ^d^ scales, mean (SD)**																												
	Anxiety		5.8 (5.3)			8.0 (5.0)			11.9 (5.5)			9.3 (6.3)		5.8 (4.9)				6.6 (5.3)			5.8 (5.6)							8.1 (5.8)
	Depression		3.9 (3.8)			3.3 (3.1)			4.1 (3.7)			4.8 (3.8)		3.5 (3.1)				6.3 (3.7)			4.4 (3.8)				4.4 (3.7)			
	Distress		19.0 (8.4)			19.2 (7.5)			20.5 (7.6)			23.6 (6.9)		21.5 (6.9)				23.7 (6.8)			19.1 (8.2)				21.5 (7.5)			
	Somatization		10.5 (6.8)			11.1 (6.6)			15.3 (6.9)			14.7 (7.4)		13.6 (6.7)				12.6 (6.9)			12.4 (7.1)				13.3 (7.1)			
**Level of care, n (%)**																												
	No care		15 (7.61)			62 (5.64)			28 (2.55)			31 (3.05)		61 (5.55)				55 (5)			13 (5.20)				265 (4.52)			
	General practice		46 (23.35)			198 (18)			165 (15)			90 (8.86)		171 (15.55)				183 (16.64)			19 (7.60)				872 (14.87)			
	Basic: short		11 (5.58)			127 (11.55)			92 (8.36)			93 (9.15)		110 (10)				102 (9.27)			8 (3.20)				543 (9.26)			
	Basic: moderate		4 (2.03)			90 (8.18)			69 (6.27)			41 (4.04)		84 (7.64)				34 (3.09)			7 (2.80)				329 (5.61)			
	Basic: intensive		23 (11.68)			340 (30.91)			340 (30.91)			244 (24.02)		457 (41.55)				283 (25.73)			29 (11.60)				1716 (29.27)			
	Specialist		98 (49.75)			283 (25.73)			406 (36.91)			517 (50.89)		217 (19.72)				443 (40.27)			174 (69.60)				2138 (36.47)			
Lexical characteristics: words (N), mean (SD)				55.1 (55.0)			71.7 (69.5)			68.0 (103.5)			75.1 (157.0)		70.9 (74.9)				65.5 (75.2)			76.4 (72.4)				69.9 (98.2)		

^a^PTSD: posttraumatic stress disorder.

^b^For patients who entered the study through their general practitioner, the gender is not registered; as such, gender is unknown for a large group of patients.

^c^DIPP: Digitale Indicatiehulp Psychische Problemen (Digital Indication Aid for Mental Health Problems).

^d^4DSQ: Dutch 4D Symptom Questionnaire. For the 4DSQ, trichotomized 5-point scale responses on each subscale are reported (see the study by Terluin et al [27] for the exact scoring method). Scores are considered moderately elevated (>10, >2, >8, >10) or strongly elevated (>20, >5, >12, >20) for distress, depression, anxiety, and somatization, respectively.

The demographic information (Table 2) shows that for those patients whose gender is known, more women than men had registered for all treatments except for Addiction. The mean age of the sample was 37.7 (SD 13.6) years, where patients treated for eating disorders were considerably younger (mean 30.8, SD 10.0 years) and patients treated for somatic disorders slightly older (mean 41.2, SD 11.7 years). The DIPP questionnaire results show that patients in treatment for panic attacks had the highest anxiety and somatization scores compared with those in other treatments. Patients treated for mood disorders scored higher on the depression and distress scale than those treated for other disorders. From the lexical characteristics, it can be concluded that the texts written by patients treated for addiction were considerably shorter: the mean number of words was 55.1 (SD 55.0) compared with an overall mean number of words of 69.9 (SD 98.2) for the complete sample. Patients with PTSD and eating disorders wrote relatively longer answers (mean 75.1, SD 157.0, and mean 76.4, SD 72.4, respectively).

### Screening Model

#### Overview

In the exhaustive grid search in the inner 5-fold cross-validation loop, all possible combinations of parameter values listed in the *Analytical Strategy* section were compared to find the model with the highest performance score. This resulted in a linear support vector classifier with a weighted *F*_1_ score of 0.471. The selected model consisted of 470 unigrams (single words) weighted by term frequency. For this model, stop words were excluded and the selected keywords had to occur in at least one of the documents in the training set. The optimal value found for the regularization parameter *C* was 1. An overview of the selected model parameters is presented in Table 3.

**Table 3:** Best parameters selected by exhaustive grid search.

Parameter	Best value
Remove stop words	Yes
Minimal *x*^a^ documents	1
Representation scheme	Unigrams
Term weight	Term frequency
Select *k*^b^ best features	470
Regularization parameter *C*	1

^a^*x*: number of documents a feature should be present in.

^b^*k*: number of most informative features selected.

#### Most Informative Features

The 50 most informative unigrams (from hereon referred to as “keywords”) are listed in Table 4. The keyword column contains the translated English keywords, followed by the Dutch stemmed keywords in parentheses. The large chi-square values and highly significant *P* values (when applying the O-a-A strategy, chi-square value *>*3.84 is required to indicate significant differences [*P*<.05]) show that there are significant differences between the observed and expected frequencies with which the keywords occur in texts written by patients with different disorders. These keywords are considered informative and were therefore included in the model. The remaining columns show the frequency with which each keyword occurs in each class (classes being the disorders for which the patients are being treated). For each keyword, the class in which it occurs most is presented in italics. This shows that especially for the eating disorder, many highly distinctive keywords were found: 22 of the 50 keywords have the highest frequency of occurrence in Eating. Some keywords have a high occurrence in several of the classes; for example, the word *fear* occurs often in the classes Panic (N=574), Anxiety (N=411), and PTSD (N=205). Of the top 50, none of the keywords occurs the most in Anxiety, and only a few have the highest occurrence in Mood and Addiction.

**Table 4:** The 50 most informative features (keywords) of the multi-class classifier with the highest chi-square values and significant (*P*<.05) *P* values.

English keyword (Dutch stem)	Chi-square (*df*)	*P* value	Addiction^a^	Anxiety^a^	Eating^a^	Mood^a^	PTSD^a,b^	Panic^a^	Somatic^a^
food (eten)	437.0 (1)	<.001	1	18	*218* ^c^	19	20	32	22
binge (eetbui)	407.3 (1)	<.001	0	3	*121*	3	3	0	2
fear (angst)	126.6 (1)	<.001	17	411	25	98	205	*574*	82
eating disorder (eetstoornis)	100.9 (1)	<.001	0	1	*33*	1	3	1	1
panic attacks (paniekaanvall)	96.6 (1)	<.001	0	13	2	12	21	*196*	11
to vomit (brak)	93.1 (1)	<.001	0	6	*28*	0	2	0	4
bulimia (boulimia)	78.4 (1)	<.001	0	1	*26*	0	0	0	0
eating pattern (eetpatron)	75.8 (1)	<.001	0	0	*24*	2	1	0	1
weight (gewicht)	69.9 (1)	<.001	0	0	*26*	4	1	0	3
to throw up (overgev)	62.2 (1)	<.001	2	16	*39*	0	1	19	4
panic (paniek)	57.7 (1)	<.001	8	42	4	22	49	*185*	23
eat (eet)	53.4 (1)	<.001	2	6	*33*	2	4	7	2
drink (drink)	48.0 (1)	<.001	*20*	5	2	8	2	9	1
eating behavior (eetgedrag)	44.4 (1)	<.001	0	0	*14*	0	0	0	0
nightmares (nachtmerries)	42.3 (1)	<.001	0	7	0	6	*78*	8	1
binge (vreetbui)	40.9 (1)	<.001	0	0	*12*	0	0	0	0
work (werk)	39.5 (1)	<.001	30	214	26	238	172	232	*531*
past (verled)	37.4 (1)	<.001	5	74	11	65	*188*	73	47
healthy (gezond)	36.8 (1)	<.001	4	21	*50*	30	17	37	20
overeating (overet)	35.6 (1)	<.001	0	0	*9*	0	0	0	0
sense (zin)	34.5 (1)	<.001	20	41	17	*198*	78	56	103
to lose weight (afvall)	30.6 (1)	<.001	2	1	*21*	6	3	3	3
eating problems (eetproblem)	30.3 (1)	<.001	0	0	*11*	0	2	1	2
scared (bang)	30.1 (1)	<.001	13	205	22	65	131	*206*	54
to attack (aanvall)	29.5 (1)	<.001	2	6	1	8	22	*74*	7
to compensate (compenser)	28.3 (1)	<.001	0	3	*11*	0	0	0	0
fat (dik)	28.2 (1)	<.001	0	3	*12*	2	3	3	1
anxious (angstig)	27.6 (1)	<.001	6	152	8	62	102	*168*	43
tired (moe)	27.2 (1)	<.001	12	66	10	145	88	66	*214*
panic attack (paniekaanval)	27.1 (1)	<.001	1	2	0	1	3	*55*	3
drug (drug)	26.3 (1)	<.001	*14*	5	3	5	4	6	3
raped (verkracht)	23.6 (1)	.001	1	2	3	0	*44*	6	4
accident (ongeluk)	23.0 (1)	.001	7	26	1	20	*87*	30	24
overweight (overgewicht)	22.9 (1)	.001	0	1	*8*	2	1	1	1
to smoke (blow)	22.6 (1)	.001	*10*	1	1	0	6	0	0
hyperventilation (hyperventilatie)	22.5 (1)	.001	2	3	0	2	4	*51*	7
tired (vermoeid)	22.5 (1)	.001	7	33	4	60	35	38	*134*
alcohol (alcohol)	22.5 (1)	.001	*15*	9	5	6	4	6	5
abuse (misbruik)	21.1 (1)	.002	5	9	0	6	*53*	6	4
obsession (obsessie)	21.1 (1)	.002	0	2	*6*	0	0	0	0
flashback (flashback)	20.7 (1)	.002	2	1	0	4	*27*	1	0
eating (eten)	20.2 (1)	.003	0	0	*5*	0	0	0	0
heavy-headed (lustelos)	20.0 (1)	.003	9	40	6	*105*	27	23	74
control (control)	19.6 (1)	.003	9	53	49	45	36	*102*	38
ate (geget)	19.3 (1)	.004	0	0	*7*	1	0	0	0
underweight (ondergewicht)	18.9 (1)	.004	0	0	*6*	1	0	0	0
nutrition (voeding)	18.9 (1)	.004	0	2	*9*	1	1	0	0
gloomy (somber)	18.6 (1)	.005	3	32	6	*112*	32	40	38
normal (normal)	18.4 (1)	.005	8	58	55	44	83	*105*	63
addictive (verslav)	17.9 (1)	.007	*10*	4	4	3	2	1	4

^a^Occurrence frequencies for each feature in each class (disorder).

^b^PTSD: posttraumatic stress disorder.

^c^The frequency for the class in which it occurs the most is presented in italics.

#### Performance Metrics

Table 5 reports the performance scores of the final model for each class. The model performs especially well in screening for eating disorders. The high precision (0.75) for this class means that 75% (41/55) of the patients whom the model classified as having an eating disorder were indeed referred to a treatment for eating disorders by the therapist. The high recall (0.82) shows that 82% (41/50) of the patients who were referred to a treatment for eating disorders by the therapist were also identified as such by the model. The model screens the least effective for addiction and anxiety. Only 25% (13/52) of the patients who were classified by the model as having an addiction and 44% (77/175) of the patients with anxiety were also identified as such by the therapist. Of the patients referred to treatments for addiction and anxiety by the therapist, respectively, only 33% (13/40) and 35% (77/220) were also found by the model. The overall accuracy of the classifier is 0.49, meaning that 49.28% (578/1173) of the predictions made by the model were correct. For a 7-class classifier this exceeds random guessing, which would be 14% (1/7).

**Table 5:** Performance metrics final model: per class and average performance scores for the final model (N=1173).

Disorder	Patients in test set, n (%)	Precision	Recall	*F*_1_ score	Overall accuracy^a^
Addiction	40 (3.41)	0.25	0.33	0.28	—^b^
Anxiety	220 (18.76)	0.44	0.35	0.39	—
Eating	50 (4.26)	0.75	0.82	0.78	—
Mood	220 (18.76)	0.44	0.50	0.47	—
PTSD^c^	203 (17.31)	0.57	0.52	0.54	—
Panic	220 (18.76)	0.57	0.55	0.56	—
Somatic	220 (18.76)	0.46	0.50	0.48	—
Weighted average	N/A^d^	0.50	0.49	0.49	0.49

^a^Accuracy is the overall accuracy of the classifier averaged over all classes.

^b^Data not available for separate classes.

^c^PTSD: posttraumatic stress disorder.

^d^N/A: not applicable.

#### Confusion Matrix

The confusion matrix in Table 6 contains the absolute counts and normalized values (counts corrected by the number of documents present in each class, in %) for the true and predicted labels. The normalized values are the most useful because these indicate the proportion of correctly predicted labels for each class, independent of the class sizes. The normalized values on the diagonal show that the classifier screens the best for Eating (41/50, 82% correct), followed by Panic (121/220, 55%), PTSD (105/203, 51.7%), Somatic (111/220, 50.5%), Mood (110/220, 50%), Anxiety (77/220, 35%), and Addiction (13/40, 32.5%). Of the 1173 patients in the test set, this screener referred 578 (49.28%) to the correct treatment.

**Table 6:** Confusion matrix for the 7-class classifier: absolute and normalized values (%) for the true versus predicted class labels.

True disorder	Predicted disorder						
	Addiction	Anxiety	Eating	Mood	PTSD^a^	Panic	Somatic
Addiction (N=40), n (%)	*13 (32.5)* ^b^	3 (7.5)	1 (2.5)	8 (20)	3 (7.5)	3 (7.5)	9 (22.5)
Anxiety (N=220), n (%)	11 (5)	*77 (35)*	6 (2.7)	33 (15)	27 (12.3)	41 (18.6)	25 (11.4)
Eating (N=50), n (%)	1 (2)	1 (2)	*41 (82)*	4 (8)	1 (2)	0 (0)	2 (4)
Mood (N=220), n (%)	11 (5)	26 (11.8)	0 (0)	*110 (50)*	14 (6.4)	10 (4.5)	49 (22.3)
PTSD (N=203), n (%)	2 (1)	18 (8.9)	0 (0)	36 (17.7)	*105 (51.7)*	19 (9.4)	23 (11.3)
Panic (N=220), n (%)	4 (1.8)	27 (12.3)	3 (1.4)	24 (10.9)	18 (8.2)	*121 (55)*	23 (10.4)
Somatic (N=220), n (%)	10 (4.5)	23 (10.5)	4 (1.8)	37 (16.8)	16 (7.3)	19 (8.6)	*111 (50.5)*

^a^PTSD: posttraumatic stress disorder.

^b^The diagonal cells show the correctly predicted labels (in italics). The off-diagonal cells show the prediction errors for each class.

**Figure 3:**
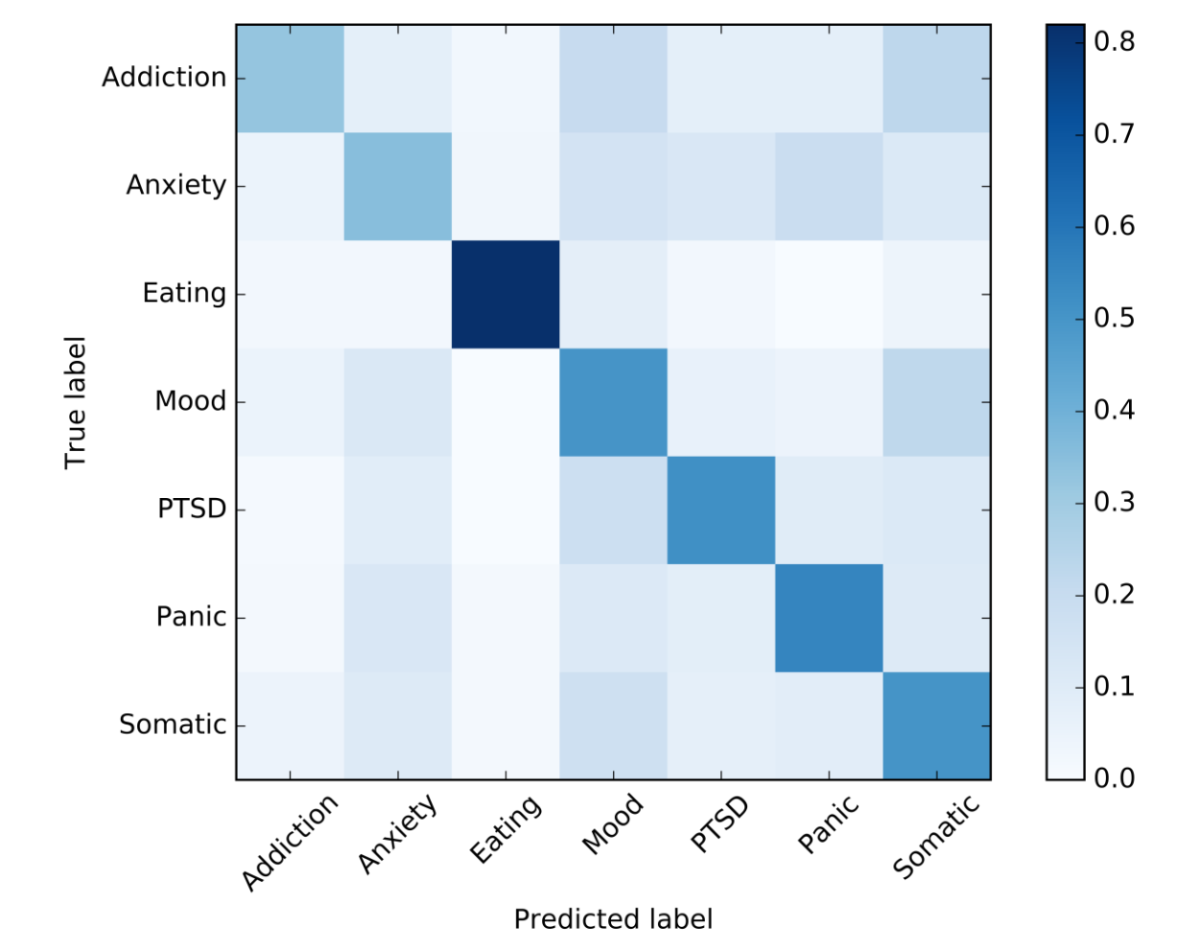
Normalized confusion plot. Visual presentation of the true versus predicted class labels. The darker the tone, the higher the proportion in the corresponding cell. PTSD: posttraumatic stress disorder.

The normalized confusion matrix is plotted in Figure 3 to give a more direct visual presentation of which classes are being misclassified. The darker the blue tones, the higher the proportions in that cell. The perfect classifier would have a dark blue diagonal line, surrounded by white cells. The plot confirms that Eating is rarely misclassified. Most confusion occurs for Addiction, which is often mislabeled as a mood or somatic disorder. In addition, mood and somatic disorders are often confused with each other, as are panic and anxiety disorders.

#### Final Model Evaluation

The 5-fold cross-validation grid search was conducted 5 times in the inner loop, iteratively using 4 of the 5 folds from the outer loop as the development set once. This resulted in 5 weighted *F*_1_ scores: one for each final model selected in the inner cross-validation loop that was tested on the test set in the outer cross-validation loop. The weighted *F*_1_ scores for the 5 outer test folds were 0.49, 0.49, 0.47, 0.46, and 0.47. The scores are relatively close to each other, meaning that the classifier generates stable results. The mean weighted *F*_1_ score over the 5 iterations was 0.48 (SD 0.01). This is the estimated generalization performance, the performance that can be expected when the final model is applied to new data sets in the future.

## Discussion

### Principal Findings

This study aims to improve the intake procedure of a web-based mental health therapy provider by using multi-class text classification to automatically screen textual answers on open questions from an intake questionnaire for a range of different mental health disorders. The resulting classification model turned out to be especially effective in screening for Eating, correctly identifying 82% (41/50) of the patients with an eating disorder. This is comparable to binary classifiers in previous studies; for example, for PTSD (80% correct; performance score for the SVM model based on unigrams) [22] or depression (84% correct) [23]. The correct classification rates for the other disorders were substantially lower: Panic, 55% (121/220); PTSD, 51.7% (105/203); Mood, 50% (110/220); Somatic, 50.5% (111/220); Anxiety, 35% (77/220); and Addiction, 32.5% (13/40), resulting in an overall accuracy of 49.28% (578/1173). This is a reasonable score for a 7-class classification model, although not high enough to make strong and accurate referrals for all treatments.

The difference in performance is also reflected in the selected keywords, of which many are highly discriminative for Eating. For example, simple words such as *food, binge, weight,* or *bulimia* are clearly related to eating disorders while sparsely being used in texts written by patients with other disorders. For the remaining disorders, the keywords found are more generally related to fears and feelings and occur more in all classes except for Eating and thus are less discriminative. For example, *fear* and *scared* are selected as keywords for Panic, but they also have high occurrences in Anxiety and PTSD. *Sense* is a keyword for Mood, but it is also highly used in texts written by patients with somatic disorders, whereas the somatic keyword *tired* is also used often in texts written by patients with a mood disorder. As a result, the model could not accurately differentiate between mood and somatic disorders as well as between panic and anxiety disorders. None of the 50 most informative keywords was related mostly to Anxiety, for which one of the lowest classification performances was reported.

The reasons for the overlap in keywords for different disorders may be symptom overlap (in case symptoms are part of the defining symptom set of multiple disorders) and nonspecificity of defining symptoms (in case symptoms also occur regularly in persons without the disorder), both issues resulting from definitional choices made in the Diagnostic and Statistical Manual of Mental Disorders, Fifth Edition [48]. For example, PTSD has overlapping symptom criteria with depression, generalized anxiety disorder, and panic disorder [49]. When (future) patients are asked to describe their most important symptoms (1 of the 3 open intake questions, the answers to which were used to develop our model; Multimedia Appendix 1), because symptoms for several disorders overlap, it is not surprising that descriptions and thus keywords for these disorders will also overlap.

The low screening performance for Addiction could be because only a very small number of patients with addiction were present in the data set (n=197), and as such the machine learning algorithm was provided with inadequate training data for this class. However, for Eating, not many more patients were included (n=250), and for this class the classifier performed very well. Another reason could be that patients in Addiction were found to write shorter texts; on average, the mean number of words used by patients in the Addiction class is 55.1 (SD 55.0) versus an average of 69.9 (SD 98.2) over all classes and even 76.4 (SD 72.4) for the Eating class (Table 2). This shows that patients with an eating disorder provide a more extensive description of their symptoms, treatment goals, and anamnesis than patients with addiction. Because of this, less information is available for Addiction than for Eating, which makes it hard for the machine learning algorithm to learn key features for this class.

The results further show that the classifier has difficulty differentiating mood from somatic disorders and panic from anxiety disorders. For mood and somatic disorders this can be explained by the fact that most patients with somatic disorders are commonly found to have an underlying mood disorder [50]. The difficulty in distinguishing between panic and anxiety disorders could be because panic disorder is actually classified as a type of anxiety disorder in the Diagnostic and Statistical Manual of Mental Disorders, Fifth Edition [51]. Despite the underlying similarity, we expected that panic disorders could be easily distinguished from anxiety disorders because of their distinctive characteristics. Although the classifier found quite a few significant keywords for Panic (eg, *fear*, *panic attack*, and *panic*), these words also occurred often in texts written by patients with Anxiety and PTSD and thus were not discriminative enough. In contrast, none of the top 50 keywords had the highest frequency of occurrence in the Anxiety class, meaning no highly discriminative keywords were found for Anxiety. As Panic and Anxiety are closely related, merging the 2 classes into one would probably improve the performance of the screener. However, this would reduce the practical applicability of the screener because the goal is to refer patients to the most suitable treatment offered by the health care provider, which offers separate treatments for Panic and Anxiety.

### Theoretical and Practical Contributions

First, this study extends the findings of previous research on text-classification applications in mental health care in that it investigates the use of a multi-class classifier instead of a binary classifier, which is predominantly used [20,21]. This way it is possible to screen for multiple disorders at once, without the need to make prior assumptions regarding the type of disorder a new patient signs up with. Second, this study shows an application of text mining and natural language processing applications originally developed for English text to non-English, in this case Dutch, mental health data. Although most of the scientific publications in this area focus on English data and tools [20,21], most underlying processes and techniques are not language dependent and as such can be easily applied to non-English texts. Finally, our data set contained high-quality class labels, consisting of official clinical diagnoses made by a therapist, enabling us to compare the labels predicted by the classifier to an official *gold standard* instead of a proxy. The quality of the labels is highly important for the performance, validity, and clinical applicability of the developed model, and acquiring large, high-quality mental health text data sets is found to be challenging [21].

For the web-based mental health provider, the developed text screener provides an additional outcome score that can be used as input for the automatically generated indicative diagnosis and for the formal diagnostic interview by the therapist. Although the overall performance of the classifier still needs to be improved, the classifier was able to distinguish eating disorders very well. As an eating disorder is currently not reported as a separate scale in the DIPP questionnaire (which reports on anxiety, depression, distress, and somatization), the text screener provides additional information that was not available from the multiple-choice questions.

This study further shows how text mining, specifically text classification, can add value to current (web-based) mental health care practice because it can be used for more efficient screening, intake, or treatment referral. As described previously, mental health problems often remain undiagnosed and untreated. This can partly be attributed to the fact that most people are only seen by primary care providers who do not always recognize mental health conditions because of comorbidity between physical and psychological diseases. Magruder et al [8] therefore propose that primary care clinicians should receive more training on the recognition of these conditions. However, even after being diagnosed, patients often remain untreated because of the scarcity of health care resources. To scale up the mental health workforce, the World Health Organization [52] has proposed to shift caregiving to mental health workers with lower qualifications or even lay helpers under the supervision of highly qualified health workers [8]. An alternative way of reducing the workload for mental health workers is to increase the use of modern technologies in screening, providing treatment, and monitoring treatment outcomes. Instead of (or in addition to) extra training for primary care providers, an automatic screening tool could also aid in the recognition of mental health problems, and instead of shifting care to lower-qualified or lay helpers, mental health providers could be supported by modern technology. The automatic screener described in this paper should be seen as an example of this.

### Limitations

An important limitation of our classifier is that it is not capable of dealing with comorbidity. Comorbidity is an important issue; 45% of the patients with psychiatric disorders are reported to meet the criteria for ≥2 disorders within the same year [48]. As stated earlier, it is not unusual for patients with somatic disorders to have an underlying mood disorder [50], whereas mood disorders are commonly found to co-occur with anxiety disorders [48]. Substance use disorders are also often found to co-occur with other mental health disorders; for drug use disorders in particular, high associations with anxiety (especially panic disorder) and affective (mood) disorders have been reported [53-55]. The main limitation of this study is that although the multi-class classifier can screen for multiple disorders at once, it does not take into account the possibility that a patient can have a combination of multiple disorders simultaneously (comorbidity). This may explain why the screener did not prove to be very capable when it came to distinguishing between some disorders, which indicates the need for a multi-label classifier that can screen for combinations of disorders instead of only a single disorder.

Another limitation may be the fact that we used a blinded tool to develop the automatic screening model. Some might state that to develop a model, at least some insight into the input data is required to actively monitor the development process. However, the tool was tested and applied in a previous study by the authors and in a master’s thesis [47] in which the process and outcomes were confirmed. This tool enabled us to work on sensitive information without any insight into the textual content, on a local computer, and without the need to send the information over the internet for processing and analysis, thereby reducing not only the risk of privacy issues, but also the risk of possible confirmation bias because of prior knowledge. However, by using a tool, one is limited by the choice of models and parameters made beforehand during the development of the tool. Adding to, or changing, the tool’s settings based on new insights is quite laborious because this requires developing, updating, and installing a new version. Therefore, we chose to use a common and proven classifier and analytic approach [21].

Yet another limitation could be the definition of the classes and class imbalance. The classes used in this study are defined by the specific diagnoses for which treatment is offered by the mental health clinic Interapy, instead of symptomatology. The performance of the classifier might be improved by grouping together comorbid disorders or disorders with overlapping symptoms (eg, combine somatic and mood disorders or panic and anxiety disorders). However, because this would decrease the practical usability of the screener, we chose to keep these classes separate. Model performance may also be influenced by class balance (or imbalance), that is, the extent to which the texts are evenly distributed across the classes. The classes Addiction and Eating were strongly underrepresented in our data set, and despite the use of class weights and stratified samples, performance for the Addiction class especially was poor. In contrast, the highest performance was reported for the Eating class; therefore, it seems that as long as the text content is discriminative enough, even small samples may provide enough information to make strong predictions.

### Future Research

Future research should focus first of all on improving the overall performance of the classifier. The current screener does not show a high enough performance for all classes, which might be solved by trying alternative classification algorithms or machine learning strategies such as a multi-label strategy to deal with comorbidity. In addition to adopting a multi-label approach, exploring a multistage learning system also seems a useful next step. Multistage models (eg, cascade classifiers) use a staged decision process in which the output of a model (the first stage) is used as the input for a successive model (the second stage), and so on. Multistage models are widely used in medical practice, and physicians use this approach for the stepwise exclusion of possible diagnoses [56]. Several studies show that multistage classifiers outperform the single-stage classifiers generally used in supervised multi-class classification tasks; for example, in the prediction of liver fibrosis degree [57] and in distinguishing among levels of dementia [56]. For our screener it could be useful to first classify the disorders into more general groups of (possibly) overlapping disorders, grouping Anxiety, Panic, and PTSD in 1 class and Mood and Somatic symptom disorders in another while keeping Eating and Substance abuse disorders separate, followed by a more specialized classification model to distinguish among the specific disorders within the groups. This prevents the best predictable class (in our case, Eating) from dominating the machine learning process. In addition, because one of the problems was finding (enough) discriminative keywords for some of the disorders, adding additional open questions to the web-based intake procedure to collect more text data may be helpful. Adjusting the questions by focusing less on symptoms (which are found to overlap for some disorders) and focusing instead on aspects possibly more defining for each disorder may also lead to more discriminative keywords and consequently better models.

Second, further uses of text mining and machine learning in mental health care practice should be explored. Text mining can be (and is) used for many more activities during and after treatment; for example, in analyzing patient–physician or patient–carer communication [58] or in evaluating treatments by capturing patients’ opinions from comments on the web [59]. In addition, text mining can also be used to assess factors and processes underlying recovery of, for example, patients with an eating disorder [60]. A new application for text mining in e–mental health practice could be to use it as a tool to support therapists by offering suggestions for patient-specific feedback. The current computerized CBT process as used in this study consists of sequential homework assignments covering common CBT interventions. On the basis of the content of these assignments, therapists offer standardized feedback and instructions, including motivational techniques, adapted to the needs and situation of the patient [14]. It would be interesting to examine whether we could use text mining to automatically highlight sections in the assignments that require attention or that may indicate a positive or negative change in behavior.

### Conclusions

This study showed that automatic text classification can improve the current web-based intake and referral procedure of a Dutch mental health clinic by providing an additional outcome score to be used as input for the indicative referral advice and the formal diagnostic interview. Automatically generating an additional indicator based on the textual input may lead to a more efficient and standardized intake process, saving time and resources because the text no longer needs to be processed and interpreted by the therapist. As such, automatic text screening could be a step in the right direction for solving patient, systemic, and provider factors underlying the underdetection of mental health disorders and underuse of available mental health treatments [6]. The overall complaint-discriminating quality of the screener still has to be improved, but the good detection performance with regard to eating disorders in this study (and with regard to PTSD and depression in other studies) shows that text-based screening is a promising technique for psychiatry. This paper contains multiple recommendations for research paths that could improve this complaint-discriminating quality of text screeners (eg, using stratified analysis techniques when symptoms overlap complaints). Altogether, the technique is getting closer to implementation in general practice, where it definitely could be of great value. Especially in areas around the world with a limited number of mental health care workers, automatic text classification could be helpful. It could save time that is now spent on screening and assessment of patients, time that could be used for counseling and treatment.

## Appendix

Multimedia Appendix 1 The translated Digitale Indicatiehulp Psychische Problemen (DIPP; Digital Indication Aid for Mental Health Problems) questionnaire used for the web-based intake of new patients. The DIPP questionnaire was originally developed and validated in Dutch [24]. The DIPP questionnaire starts with the Dutch version of the 4D Symptom Questionnaire [26-28], followed by additional questions regarding current symptoms, treatment goals, anamnesis, psychosis risk, substance use, and medication.

## Abbreviations

CBT: cognitive behavioral therapy
DIPP: Digitale Indicatiehulp Psychische Problemen (Digital Indication Aid for Mental Health Problems)
FN: false negative
FP: false positive
O-a-A: one-against-all
PTSD: posttraumatic stress disorder
SVM: support vector machine
TN: true negative
TP: true positive
