# Supplementary material for: Improving Web-Based Treatment Intake for Multiple Mental and Substance Use Disorders by Text Mining and Machine Learning: Algorithm Development and Validation
Source: JMIR Ment Health. 2022 Apr 11;9(4):e21111. doi: 10.2196/21111 (PMC9039807; doi:10.2196/21111)
Supplement: Multimedia Appendix 1 [file mental_v9i4e21111_app1.docx]

# Multimedia Appendix: Translated DIPP questions

This Multimedia Appendix provides the DIPP questionnaire used for the online intake of new patients. The DIPP (Digital Indication Aid for Mental Health Problems) is originally developed and validated in Dutch [24]. A translation of the DIPP version that was used in this study is listed below for illustrative purposes. The DIPP starts with the Dutch version of the 4DSQ [26-28], followed by additional questions regarding current symptoms, treatment goals, anamnesis, psychosis risk, substance use, and medication. The questionnaire ends with the collection of demographic information and contact data required to schedule an appointment for the indication interview.

This is a Multimedia Appendix to a full manuscript published in the J Med Internet Res. For full copyright and citation information see http://dx.doi.org/10.2196/21111

## 4DSQ questions

First the 50 questions of the Four-Dimensional Symptom Questionnaire (4DSQ) are completed. The specific questions can be found in previous publications [26-28].

## Additional DIPP questions

### Open questions used for text screening

*Can you briefly describe your main symptom(s)?*

*What would you like to achieve with a treatment?*

*Have there been any events (such as a divorce, loss of job, or accident) that, in your opinion, affect your current symptoms, and if so, what are they?*

### Multiple choice questions

*Have there been any previous times in your life when you had similar symptoms?*

*o Yes*

*o No*

*Have you recovered from these complaints in the meantime?*

*o Yes*

*o No*

*o Does not apply*

*How long have you been on sick leave because of your current symptoms?*

*o I am not on sick leave; I do not have a paid job*

*o 1 week*

*o 2 weeks*

*o 3 weeks*

*o 4 weeks*

*o More than 4 weeks*

*How many hours are you actually working per week now?*

*I am currently working ... hours a week*

*How many hours of work per week are laid down in your employment contract?*

*... hours a week*

*Do you avoid daily, necessary activities or situations because of your symptoms (e.g., shopping, travelling by public transport, visiting friends)?*

*o Yes*

*o No*

*For how long have you been suffering from your current symptoms?*

*o One month or less*

*o 2 months*

*o 3 months*

*o 4 months*

*o 5 months*

*o 6 months*

*o More than 6 months*

*Have you previously had treatment for the same symptoms (e.g., by a psychiatrist, psychologist, general practice based mental health nurse specialist (POH-GGZ), or general practitioner)?*

*o Yes*

*o No*

*Was there a period after the treatment in which you were free of symptoms?*

*o Yes*

*o No*

**Psychosis risk**

*Have you experienced any of the following in the past five years?*

*I sometimes get messages from voices in or near my head.*

*o not at all*

*o a little*

*o quite*

*o certainly*

*o very much*

*I sometimes hear voices that other people cannot hear.*

*o not at all*

*o a little*

*o quite*

*o certainly*

*o very much*

**Substance use**

*Do you use alcohol?*

*o No*

*o Not any more, quit*

*o Yes*

*If you quit, since when? ….*

*Do you use soft drugs?*

*o No*

*o Not any more, quit*

*o Yes*

*If you quit, since when? ….*

*Do you use hard drugs?*

*o No*

*o Not any more, quit*

*o Yes*

*If you quit, since when? ….*

**Medication**

*Participants are asked about medication use;*

- *drug name*
- *dose*
- *first use*

**Date of birth**

*What is your date of birth? (dd-mm-yyyy)*

**Sex**

*What is your gender?*

*o Female*

*o Male*

**Telephone number**

Required to make a callback appointment.

**Schedule an appointment**

When all questions have been answered, there are two options to schedule an appointment for the indication interview; this depends on the practice/organization that applies the DIPP:
A. the patient can choose from a number of day times offered in the application;
B. the patient provides his telephone number and is called by the assistant or secretary for an appointment.
